# *Aureococcus anophagefferens* strain CCMP1851: draft genome of a second *Kratosvirus quantuckense-*susceptible host strain for an emerging host–giant virus model system

**DOI:** 10.1128/mra.00292-24

**Published:** 2024-05-03

**Authors:** Emily E. Chase, Alexander R. Truchon, William W. Schepens, Steven W. Wilhelm

**Affiliations:** 1 Department of Microbiology, University of Tennessee, Knoxville, Tennessee, USA; Rochester Institute of Technology, Rochester, New York, USA

**Keywords:** harmful algal blooms, *Nucleocytoviricota*, brown tides, giant viruses, Pelagophyceae

## Abstract

Here, we report the draft genome of *Aureococcus anophagefferens* strain CCMP1851, which is susceptible to the virus *Kratosvirus quantuckense*. CCMP1851 complements an available genome for a virus-resistant strain (CCMP1850) isolated from the same bloom. Future studies can now use this genome to examine genetic hints of virus resistance and susceptibility.

## ANNOUNCEMENT

We assembled a draft genome for an isolate of the marine algae *Aureococcus anophagefferens* (Pelagophyceae) (1). *A. anophagefferens* strain CCMP1851 is only the second strain with a genome made public that can be infected by the “giant” virus *Kratosvirus quantuckense* (*Nucleocytoviricota*) (2–5). It was isolated from the same bloom as the strain CCMP1850 (September 1998, Great South Bay, Long Island; NCMA, Bigelow), which is resistant to this virus (4, 5). This resistant–susceptible pair from a similar evolutionary time enables in-lab genomic comparisons of susceptible and resistant hosts. We acknowledge the natural biology of the system, which enables single-strain monocultures deemed as “resistant” and “susceptible” to include individual cells that do not meet the requirements of “resistant” or “susceptible,” and that host resistance may occur in different ways and to different degrees. Nonetheless, this genome represents insight into two strains within a single bloom with different interactions (resistance or not) to contact with *K. quantuckense*.

Cells were propagated at 19°C on a 12/12 light cycle (75 µmol·m⁻²·s⁻¹). Nucleic acid extraction used phenol–chloroform (6) without lysozyme. Unless stated, all *in silico* analyses used default parameters. Long-read sequencing used the Oxford Nanopore Technologies (Oxford, UK) platform (ligation on MinION R9.4 flow cell), producing ~4.96 million reads (N50 = 6.05 kbp, maximum read length = 87,550 bp) (Table 1). Bases were called with Guppy basecaller v.6.5.7+ca6d6af, adaptors removed with Porechop v.0.2.4, and reads filtered using NanoFilt v.2.8.0 based on a read length >500 bp and quality score of 9 (2, 7, 8). Short reads were sequenced (SeqCenter, Pittsburgh, PA, USA) on a NovaSeq 6000 (150 bp paired-end; ~21.6 million reads) using a Nextera DNA Flex Library Prep. Short reads were trimmed using Trimmomatic v 0.39 (9). An assembly incorporating short and long reads was generated through Unicycler v.0.5.0 (10) with Racon (11). Redundans v2.01 (12) was used to remove redundant/heterozygous contigs. Circular contigs (predicted by Unicycler) around the expected size range of chloroplast and mitochondrial genomes were recovered and annotated with Prokka v1.14.6 (13). Contaminants were removed by splitting all contigs into 1,000 bp windows and running each through Kaiju v1.10.0 (14), and contigs with a majority rule hit to *A. anophagefferens* were maintained. Coding sequences and tRNAs were recovered with MAKER v.3.01.03 (5, 15) and tRNAscan-SE v2.0 (16) and annotated using eggNOG v.6.0 (17). BUSCO v5.2.2 (18) was run to check completeness (91%). Single-copy orthologous genes among publicly available *A. anophagefferens* genomes were recovered using OrthoFinder v2.5.5 (19) with *Pelagomonas calceolata* as an outgroup (NCBI accession CAKKNEO1; Pelagophyceae). All resulting single-copy orthologs (*n* = 1,272) were aligned using MAFFT v7.520 (20) and trimmed using trimAL v1.4.rev15 automated1 (21). A phylogeny was produced using IQ-TREE v2.2.2.3 (22) with a built-in ModelFinder test and visualized using iTol v5 (23).

**TABLE 1:** Sequencing statistics and general assembly information of *Aureococcus anophagefferens* strain CCMP1851

Attribute	Information
Assembly N50	424,265
Assembly L50	40
Total assembled contigs	1,563
Contigs over 1,000	1,417
Genome scaffolds	461
Genome size	49,916,805 bp
GC content	70.5%
CDS count	13,566
rRNA count	4
tRNA count	123

**Fig 1:**
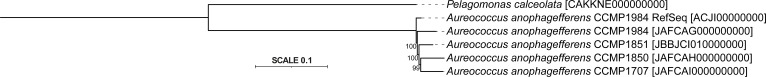
Phylogenetic relationships among *Aureococcus anophagefferens* strains (WGS NCBI: ACJI00000000, JAFCAG000000000, new strain CCMP1851; JBBJCI010000000, JAFCAH000000000, JBBJCI010000000, and JAFCAI000000000), with a more distantly related outgroup (CAKKNE000000000). Bootstrap replicates are indicated at tree nodes (*n* = 1,000). A VT+F+G4 (i.e., variation and transition substitutions, equal frequency among nucleotides, and a gamma distribution rate with four categories) model was chosen by ModelFinder (via BIC). The tree is based on an alignment of 613,029 amino acid positions.

Interestingly, the single-copy orthologous gene tree indicated a closer evolutionary relationship between CCMP1850 and CCMP1707 (Fig. 1), suggesting a stronger similarity among resistant strains despite being isolated three years apart. Future work will focus on unique genes between CCMP1851 and CCMP1850 to give context to virus resistance versus virus susceptibility to *K. quantuckense*.

## Data Availability

The raw sequencing reads and the assembled genome have been indexed at NCBI under the BioProject accession number PRJNA1047062. The assembled genome has been assigned as WGS JBBJCI010000000. The Illumina and Nanopore sequencing reads have been submitted to the Sequence Read Archive under the accession numbers SRR27225910 and SRR27225911.
